# Contribution to the Etiology of Lameness in Colts

**Published:** 1898-03

**Authors:** M. Caspar


### ABSTRACTS FROM FOREIGN JOURNALS.

### GERMAN.

Under the Direction of J. Preston Hoskins, Ph.D.,
PRINCETON, N. J.

Contribution to the Etiology of Lameness in Colts. By M. Caspar.—An article in the *Monatsheften für Thierheilkunde*, vol. viii., No. 6, by Gmelin, entitled "A Contribution to the Knowledge of Infectious Inflammation of the Navel in Calves and Colts," has led me to publish here the result of the bacteriological investigations which I made in 1893, at the Pathological Institute of the Berlin Veterinary High School, in reference to two cases of lameness in colts.

I.—On April 5, 1893, a colt, four days old, which had died the day before, was brought to the Pathological Institute. Anatomical condition : The colt was lean and lank, the muscles flabby. The orifice of the navel was closed, the stump of the umbilical cord colored black, hard, dry, and rough. In the free space of the abdominal cavity about one-half litre of a yellowish-red and slightly muddy fluid was found. The position of the intestines was normal ; the intestines were pale ; the mucous membrane of the stomach and intestines without especial deviations. The umbilical vein was noticeable, like a bluish-red cord, about as thick as the little finger, but the breadth was not the same in the different sections of it. Close beside it in the peritoneum numerous fine blood-spots about as big as a pinhead could be noticed. These were also visible at other places on the abdominal wall. The umbilical vein was filled almost to bursting with blackish-red blood, which for the most part had coagulated. At the outer end the wall of the vein attained the circumference of a lentil, and was grayish-red and cloudy in color, but with no overlayer worthy of mention. The umbilical arteries were about as thick as a lead-pencil, and bluish-red, decreasing in size as they approached the aorta. Their contents consisted of blackish-red blood almost entirely coagulated. The liver was large, the left lobe about as large as the right, the whole bluish-red and firm. A section of the same was brownish-red. The outlines of the acini were hardly recognizable. The spleen was about 24 cm. long, 12 cm. broad, and 2½ cm. thick, grayish-blue in color, the surface slightly granulated, the edges rounded, the capsule stretched, and a section brownish-red in color. The kidneys were grayish-brown and smooth. The central layer of a section was pale, yellowish-white; the outer layer grayish-brown. On the front and back surfaces of the diaphragm numerous spots of blood were visible, some shaped like a point, and others more elongated. The lungs were very large and brownish-red. Different parts, especially on the front lobe, were free of air, somewhat sunk in and bluish-red in color. The section of the lung in general was brownish-red, smooth, and the tissue was œdematously saturated. Both chambers of the heart were filled with black blood, for the most part coagulated. Beneath the epicardium several bloodspots about the size of a pin-head could be seen. Beneath the endocardium these spots were more extended, appearing in patches. In the joints no changes were visible.

Bacteriological condition: In the blood of the umbilical vein, of the heart, and in the spleen numerous microörganisms were visible, of which two kinds could be distinguished. The one (cocci) were round or oval, often lying together in pairs with their long sides parallel. The others (bacilli) had the clear rod-shape, were somewhat longer than those of the swine-pest, had rounded ends, and were partly without color in the middle.

The following cultures were prepared with great care: Four agar tubes from the blood of the umbilical vein; four agar tubes from the umbilical vein of the heart; four agar tubes from the spleen; two gelatin tubes from the blood of the umbilical vein; two gelatin tubes from the umbilical vein of the heart; two gelatin tubes from the spleen; two gelatin plates were covered with blood from the heart. At the same time three mice were subcutaneously injected with small pieces of the spleen, about the size of a millet-grain. After twenty-four hours there was no sign of development on the gelatin plate nor in the gelatin tubes. On all the agar tubes shining, grayish-white colonies about the size of a lentil developed. The edges of these were notched, and the centre was raised and could be easily separated. The colonies were separated from each other. Between them were observed on the surface very small, transparent, drop-like colonies lying close to each other, and not exceeding a pin-head in size. The small colonies consisted of cocci which were often wreathed into each other. The large colonies consisted of the little rods with rounded ends described above, and could be easily colored. They were about three times as long as broad, and lay separately and in pairs.

When they were colored with methylene-blue the middle often remained colorless, and they resembled the appearance of the septicæmia-hemorrhagica bacteria. From one of the large colonies three agar plates were covered. In twenty-four hours plates 1 and 2 were so thickly sown with colonies that they ran into each other. On plate 3 separate circular colonies with notched edges, grayish-white, and not quite the size of a lentil, were visible. They consisted of the little rod-like organisms mentioned above.

The mice that were subcutaneously injected became sick after twenty-four hours. Their hair bristled up, respiration was increased, and their eyes became sticky. While two recovered during the following days, one of them died forty-four hours after infection. An examination showed the eyelids sticky, with a mattery secretion. No fluid in the abdominal cavity. Peyer's glands were dark-red and swollen, the follicle in them grayish-white and as large as a pin-head, the capillaries filled to bursting, but otherwise nothing abnormal in the intestines. The spleen was at least three times its normal size, bluish-red, with the edges rounded. The liver very large, dark-red; on the upper surface numerous little gray-white prominences could be detected. Kidneys hardly enlarged, bluish-red. The lungs grayish-red and œdematous. The heart filled with blood. In the blood there were comparatively many round and oval cocci, mostly lying in pairs, but here and there arranged in chains. On coloring them doubly with eosin and methylene-blue, the cocci appear blue surrounded by a pretty large bright-red halo or ring. This ring can be seen in the blood-preparations also, which are simply colored with methylene-blue. In the spleen these same formations were found very numerous, in pairs and in shorter chains. In the grayish-white places of the liver similar microörganisms were found; likewise in Peyer's glands, and in the lungs. These cocci are neither morphologically nor biologically distinguishable from the streptococcus pyogenes, and must be regarded as identical with the same.

II.—On April 11, 1893, there was sent to the Pathological Institute a colt, three weeks old, which had been dead a few hours. The examination, made immediately, showed the following: A strong colt, with powerful muscles. At the navel an opening about the size of a pea was noticed. The edges of this were dirty and sticky. With pressure a drop of mattery fluid oozed out. The under skin of the right fore-and hind-thigh was swollen, with a watery and bloody infiltration, muddy, and about the consistency of gall. The intermuscular tissues were likewise affected (œdema phlegmonosum). In the abdominal cavity the contents were normal, as well as the position of the viscera. The intestines on the outside showed a grayish-white to a grayish-yellow color. The mucous membrane of the digestive channel was pale, without especial changes. The umbilical vein was as thick as one's finger, bluish-red and fluctuating to the touch. It contained an offensive, grayish-brown fluid in which small particles of solid matter were noticeable. The inner wall of the vein was a dirty grayish-white. The same contents and condition of the inner wall could also be traced as far as the veins of the liver. The umbilical arteries were bluish-red, as thick as a quill, and in the neighborhood of the navel contained a muddy, grayish-red fluid. Further on, blood, mostly coagulated, was found. The liver was large, bluish-red, and muddy; the section was bloody, brownish-red, with the marking of the acini unclear. The spleen was 39 cm. long, 17 cm. broad, and 2 cm. thick, grayish-blue, and hard. The surface was slightly uneven, the edges not sharp, and a section brownish-red. The kidneys showed brownish-red through the capsule. They were smooth and enlarged; the central layer was a pale yellowish-white, the outer layer grayish-brown, muddy, and somewhat broadened out. In the sacs of the abdominal wall a large quantity ($\frac{1}{2}$ litre) of grayish-red fluid. The lungs very large, œdematous, and brownish-red. The pleuræ were smooth, slimy, and moist. Both chambers of the heart filled with mostly coagulated blood. Subendocardial bloodspots noticeable. The capsules of the joints of the right fore- and hind-thigh were swollen and red. The synovia increased, of a yellowish-red color and of a muddy consistency. The marrow of the femur, humerus and radius was dark-red both in the diaphysis and the epiphyses. The hollow in the bone was comparatively small, shaped like a slit, and filled with thick blood.

Bacteriological conditions. In the mattery contents of the umbilical vein numerous cocci (streptococci) and rod-like bacteria were found. In the blood of the heart, in the spleen, in the marrow, and in the fluids at the joints only cocci, singly, in pairs, and in chains, could be detected.

The following cultures were made from the contents of the joint of the right ankle-bone: Three agar tubes, three glycerin-agar tubes, three blood-serum tubes, and three gelatin tubes. The same number of cultures were made of the blood from the heart. At the same time four mice were subcutaneously injected with fluid from the joints and blood from the heart. The first mouse received $\frac{1}{3}$ gill of fluid from the joints; the second, $\frac{2}{3}$ gill; the third, 1 gill; and the fourth, $1\frac{1}{3}$ gills of blood from the heart.

After twenty-four hours in a breeding temperature only a very slight development could be noticed on the agar, glycerin-agar, and serum tubes. On the agar tube, after forty-eight hours, a fine thin coating was recognizable, consisting of the smallest gray-ish-white points. Microscopically, these consisted of little cocci which lay singly and in pairs. In the glycerin-agar tube the same development of little cocci, but somewhat more pronounced. In the blood-serum tube the same development as in the agar. In the gelatin tube nothing took place.

All the mice that were subcutaneously injected on April 11th became sick after twenty-four hours, showing bristling hair and sticky eyes. Three of them recovered the following days, but the one which had received $1\frac{1}{3}$ gills of the blood from the heart died after forty-eight hours. The post-mortem examination showed on the under edge of the liver a colony about the size of a pin-head, in which were many little cocci; also in the rest of the liver-tissue the same cocci, singly and in pairs. The spleen was somewhat enlarged; in the tissue many little cocci; in the blood only a few cocci could be found. From the blood of the heart of this mouse four grape-sugar bouillon tubes were prepared. In this bouillon, after two days, a strong crumby sediment formed, which became cloudy when shaken. Masses of cocci were present in it, arranged almost entirely in short chains. Here we had to do with strep-tococci in a pure culture.

In two cases of colt-lameness I have succeeded in showing in the blood and organs the presence of a microörganism—once in pure culture, the other time associated with another—which must be designated as the streptococcus pyogenes. In regard to the other species, which I did not regard further at the time, it is now clear to me that I had before me the bacterium coli commune. Lately I have made some interesting investigations in regard to the pres-ence and the significance of this bacterium in animals, and from a comparison with the permanent preparations which I made at that time, and from the manner and form of their growth, I have now no doubt that I had to do with this microörganism. That the same was found in the blood and organs only in the first case, and not in the second, is explained by the fact that the post-mor-tem on the first colt was not made until twenty-four hours after death, while in the second it was made immediately.

In the first case there was ample time to enable the coli bacterium normally arising in the intestine to get into the blood-channels. In each case only a subsidiary role can be attributed to it, as it was only present in one case.

As regards the streptococcus pyogenes I am very much inclined, in etiological connection, to ascribe great significance to it. The metastatic mattery processes in the joints can be, without difficulty, attributed to the working of this microörganism. However attractive this explanation may be, nevertheless I am not in a position to designate it as the original cause of lameness in colts so long as no one has succeeded in producing the disease by experiments which shall follow the natural method of infection as closely as possible. I was at the time not in a position to make such experiments on colts, and realize this deficiency in my proof very well.

My investigations have shown clearly that in two plain cases of lameness in colts bacteria other than the septicæmia-hemorrhagica group were not present. The presence of the same could not have escaped me in the microscopic investigation, in the cultures, and in transferring them to mice. Gmelin, as appears in the aforementioned article, found, in the infectious inflammation of the navel in calves, bacteria which, according to their morphological and biological character, must be reckoned to the group of septicæmia-hemorrhagica bacteria. Gmelin leaves the question open whether these bacteria are the only cause of the diseases grouped commonly under the head of lameness, and demanded further investigation. This demand led me to publish the observations which I had made four years ago.

So much is certain: That the result of Gmelin's investigation must not be generalized, and the conclusions which he has drawn cannot at this time be applied to all cases of this peculiar disease. I consider it going too far to apply the conclusion which Gmelin had reached in calves to the cases of colts simply on the basis of agreement in clinical, anatomical, and microscopical results. I am not convinced that he has succeeded in proving the bacteria of septicæmia hemorrhagica present in the lameness of colts.

The state of the question is therefore this at present: Gmelin, in the infectious inflammation of the navel in calves, has established the presence of bacteria which, in accordance with their morphological and biological characteristics, belong to the group of septicæmia hemorrhagica, while in two cases of lameness in colts I have shown the streptococcus pyogenes to be present.

It is quite striking that members of the profession who have plenty of material have made so few bacteriological investigations in regard to this. It would be, I think, no very difficult task to clear up the etiological side of this disease by experiments in transferring the disease. I have never had the opportunity of seeing another case of lameness in colts, yet this must often occur on large stud-farms. In the veterinary literature of the last ten years I have been able to find nothing, except Gmelin's article, which gave any definite information in regard to the nature of the specific microörganism. That the disease is of an infectious nature no one can seriously doubt from the present point of view of science.—*Deutsche Thierärztliche Wochenschrift*, May 8, 1897.

